# Retraction: High Mobility Group Box-1 Promotes the Proliferation and Migration of Hepatic Stellate Cells via TLR4-Dependent Signal Pathways of PI3K/Akt and JNK

**DOI:** 10.1371/journal.pone.0216942

**Published:** 2019-05-09

**Authors:** The PLOS ONE Editors

After publication of this article [1], concerns were raised about results reported in Figs 2 and 3:

- Data in lanes 1, 2 of the p-Pl3K blot in Fig 2A appear similar to data in lanes 2, 3 of the PI3K blot in Fig 2B.
- Similarities were noted between bands in lanes 1, 2 of the PI3K blot in Fig 2B.
- The p-JNK western blot in Fig 2A appears similar to the NF-kB blot in Fig 3A.

The authors commented that errors had been made in preparing Figs 2B and 3A, and that the panels shown in Fig 2A are correct. The authors provided a replacement image for the PI3K panel in Fig 2 and replication data for the results shown in Figs 2B and 3A. The data supplied do not address the concerns identified. A member of *PLOS ONE*’s Editorial Board advised that the replication data do not report consistent results, and do not suffice to support the article’s conclusions regarding the effects of HMGB1 and anti-TLR4 on PI3K and JNK signaling and NF-kB activation. Original uncropped image data underlying the published results are not available.

In light of the above concerns, the *PLOS ONE* Editors retract this article.

All authors agreed with the retraction.
